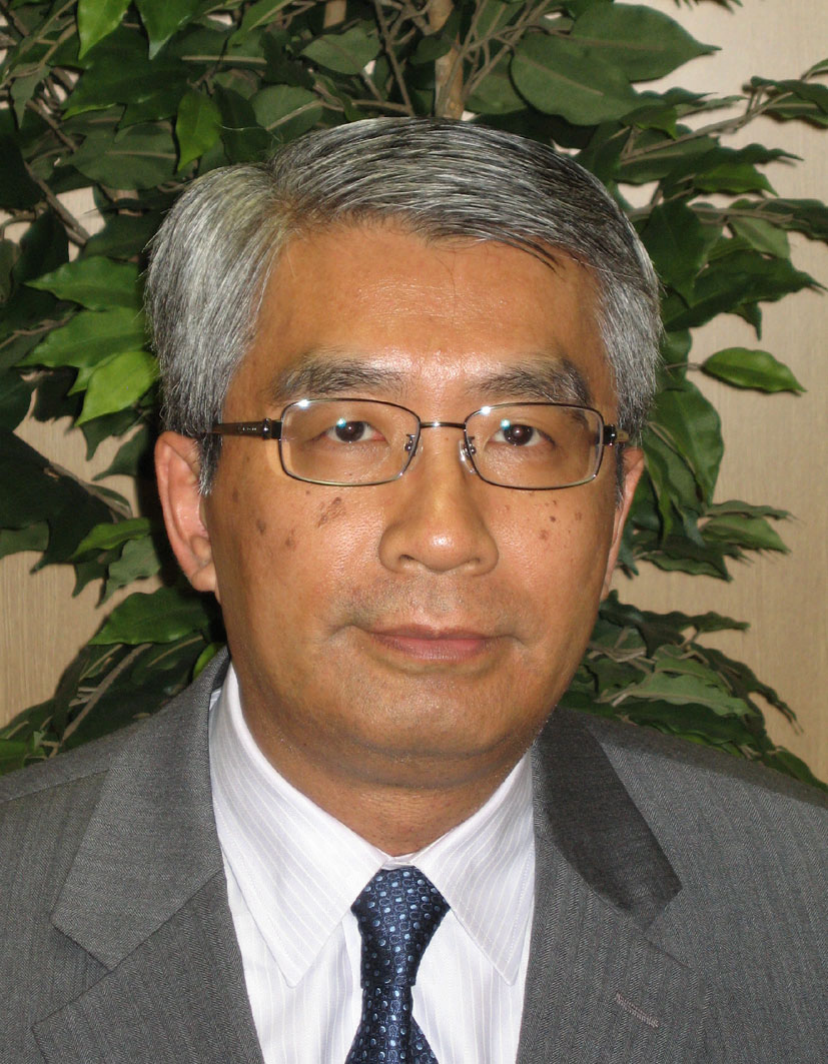# Message from the New Editor-in-Chief

**DOI:** 10.2188/jea.JE20100172

**Published:** 2011-01-05

**Authors:** Hiroyasu Iso

It is my great privilege to take over as Editor-in-Chief of the Journal of Epidemiology after Dr. Tomotaka Sobue, who served in that role between 2008 and 2010. Under the outstanding leadership of Dr. Sobue and his team, an online submission system was started in 2008, and the number of manuscripts from abroad has substantially increased. In 2010, 40% of submitted articles were from Japan, 12% from Europe, 11% from China, 9% each from Taiwan and countries in the Middle East, 5% from Korea, 6% from other Asian countries, 6% from the United States, and 2% from South America. The average impact factor from 2007 through 2009 was 1.73.

The journal will continue to cover a broad range of high-quality epidemiological research throughout the world and to contribute to the basic, clinical, public health, and political sciences. The editorial team seeks to maintain our high international standard by publishing original and review articles on a variety of research topics in order to eventually establish us as one of the leading journals in epidemiology.

Hiroyasu Iso, MD, PhD, MPH Editor-in-Chief, Journal of Epidemiology

Professor of Public Health Department of Environment and Social Medicine Osaka University Graduate School of Medicine

**Figure:**